# A semi-qualitative study of attitudes to vaccinating adolescents against human papillomavirus without parental consent

**DOI:** 10.1186/1471-2458-7-20

**Published:** 2007-02-09

**Authors:** Loretta Brabin, Stephen A Roberts, Henry C Kitchener

**Affiliations:** 1 Academic Unit of Obstetrics & Gynaecology, Research floor, St. Mary's Hospital, Whitworth Park, Manchester, M13 0JH, UK; 2 Biostatistics Group, Division of Epidemiology and Health Sciences, University of Manchester, M13 9PT, UK

## Abstract

**Background:**

The first vaccine to prevent human papillomavirus (HPV) and cervical cancer has been licensed, and in future, vaccination may be routinely offered to 10–14 year old girls. HPV is a sexually transmitted virus and some parents may refuse consent for vaccination. Under-16s in the UK have a right to confidential sexual health care without parental consent. We investigated parents' views on making available HPV vaccination to adolescent minors at sexual health clinics without parental consent.

**Methods:**

This was a semi-qualitative analysis of views of parents of 11–12 year old school children collected as part of a population-based survey of parental attitudes to HPV vaccination in Manchester. Parents were firstly asked if they agreed that a well-informed child should be able to request vaccination at a sexual health clinic without parental consent, and secondly, to provide a reason for this answer. Ethical perspectives on adolescent autonomy provided the framework for descriptive analysis.

**Results:**

307 parents answered the question, and of these, 244 (80%) explained their views. Parents with views consistent with support for adolescent autonomy (n = 99) wanted to encourage responsible behaviour, protect children from ill-informed or bigoted parents, and respected confidentiality and individual rights. In contrast, 97 parents insisted on being involved in decision-making. They emphasised adult responsibility for a child's health and guidance, erosion of parental rights, and respect for cultural and moral values. Other parents (n = 48) wanted clearer legal definitions governing parental rights and responsibilities or hoped for joint decision-making. Parents resistant to adolescent autonomy would be less likely to consent to future HPV vaccination, (67%) than parents supporting this principle (89%; p < 0.001).

**Conclusion:**

In the UK, the principle of adolescent autonomy is recognised and logically should include the right to HPV vaccination, but this may concern parents who would otherwise approve vaccination.

## Background

The first vaccine to prevent cervical cancer was recently approved for use in the European Union (Gardasil^®^, Merck &Co, Inc.)[1]. This brings closer the possibility of routine vaccination against human papillomavirus (HPV) to prevent pre-cancerous cervical lesions and invasive disease [2]. This quadrivalent vaccine, which is highly effective against HPV types 6/11 (low risk) and HPV types 16/18 (high risk), could have a significant public health impact in reducing HPV prevalence, persistent HPV infection, cervical neoplasia and genital warts [3]. A bivalent vaccine (Cervarix^® ^GlaxoSmithKline) with similar levels of protective efficacy against HPV types 16/18 is expected to be licensed in 2007 [4]. The United States Centers for Diseases Control has already recommended routine vaccination of Gardasil^® ^for 11 and 12 year old girls, and 9–10 year olds at the discretion of their physician [5]. (Pre)-adolescents are the main target group as the vaccine is prophylactic and will be most effective if administered prior to sexual initiation [6]. The public health impact will be greatest if a high coverage of the target population is achieved [7]. School vaccination programmes would be feasible in the UK but acceptability by the public of routine vaccination against a sexually transmitted infection (STI) is still uncertain and parental consent for vaccination would be necessary.

A number of studies of parental attitudes – mostly conducted in the US – suggest a considerable interest in adolescent HPV vaccination, with most parents in favour of protecting their children [8]. We previously reported a population-based survey in Manchester, UK, that assessed the acceptability of HPV vaccines to parents of 11–12 year old school children [9]. The study included parents from a wide range of backgrounds, and 81% replied that they would consent to HPV vaccination for their child, although only 38% were definite in this view. Long-term safety of the vaccine was an important issue, and a minority of parents would refuse on grounds that vaccination could encourage early sexual debut or riskier sexual behaviour. In view of the fact that, in the UK, at least 25% of adolescents will have their first experience of sexual intercourse before the age of 16 [10], and to ensure a high population coverage, it may be necessary for HPV vaccines to be available for under-16s without parental consent [11]. In the UK, vaccines could be given in general practice, at sexual health clinics, or within targeted "catch-up" programmes for women up to 26 years.

Consent for treatment is based on the ethical principle of patient autonomy, a concept that has many definitions [11,12] but is associated with the right to privacy and confidentiality, acting according to one's own volition, self-mastery, choosing one's own moral responsibility and accepting responsibilities for one's choice. Consent is valid if it is voluntary and the individual is both informed and competent [13]. In the face of increased sexual and reproductive health care needs of adolescents, many countries have enacted "health services to minors" legal Acts, lending support to a notion of "adolescent autonomy" [14]. In England the Law Lords ruled in 1985 that a girl under 16 could consent for contraception if able to understand the proposed treatment and it implications [15]. Subsequently, the Fraser ruling provided guidance for health workers on assessing the maturity and competency of a minor to comprehend the information given [16]. As HPV is sexually transmitted, it would be logical to make HPV vaccination routinely available to sexually active minors whose parents had refused consent. This could, however, stigmatise HPV vaccination and make it more contentious, as some parents are opposed to confidential sexual health services for minors [17]. To explore this issue our survey included two items, the first asking if parents would approve a well-informed child being allowed to access the vaccine at a sexual health clinic without parental consent, and the second to explain in their own words the reason for their answer. In this paper we report a semi-qualitative analysis of their responses, using an ethical framework to explore parents' views on HPV vaccination in the context of adolescent autonomy.

## Methods

### Study Procedures

The data described here are semi-qualitative, cross sectional descriptive data. The methodology and results for the main study, which was designed to sample randomly parents of year 7 pupils (ages 11–12) in the city of Manchester, UK, have been reported in full elsewhere [9]. In brief, all 26 inner-city community (state), voluntary-aided (faith-based) and independent (private) secondary schools were stratified into eight strata according to school type and ethnicity, based on data supplied by the Department for Education. Using a purpose-written computer program, one school was randomly selected from each stratum, with alternative second and third choice schools available in the event of refusals This gave a potential sample of about 1500 pupils and allowed sampling across all school types. A questionnaire, eliciting basic socio-economic data and parents' views on HPV, was developed through group discussions and subsequent piloting with parents, then mailed through the school administration directly to parents. Parents were advised that return of a completed questionnaire signified written consent to the study. Ethnic response categories were those defined in the 2001 census. Religion was categorised as follows: None, Christian (Roman Catholic), Christian (Protestant; state denomination), Muslim, Hindu, Buddhist, Sikh, Jewish, Other. As knowledge about HPV was low, key facts were given about HPV and cervical cancer in the preamble to the questionnaire, and a separate information leaflet was provided. Despite a response rate of 22% (n = 317), the major social and ethnic groups were well represented, as were religious views – except for those of one non-participant school, which was the only one of its type and could not be replaced. HPV vaccine acceptance did not vary significantly across socio-demographic groups.

The University of Manchester Committee on the Ethics of Research on Human Beings approved the study.

### Data analysis

Responses to the question of whether a well-informed child should be able to request vaccination without parental consent at a sexual health clinic were measured using a five-point Likert scale (strongly agree, agree, disagree, strongly disagree, don't know). For the detailed comments, we followed a recommended method for analysis of semi-qualitative survey data [18]. Comments were entered verbatim on to computer and sub-categorised into three groups representing parents giving positive, less positive or ambiguous responses. The numbers for each group are discrete as viewpoints generally fell clearly into one of the groups. Within each group, the responses were classified into sub-themes related to ethical principles (such as privacy and confidentiality, informed consent, maleficence and beneficence) and comments illustrating these were extracted for quotation. Parents received no guidance on the definition of a "well-informed" child. The categories were independently assigned by two researchers and discrepancies were reviewed and jointly agreed. To assess the background of parents with different viewpoints, the child's ethnicity, religion, entitlement to free school meals and parental age were cross-tabulated against these categories and compared using Pearson's chi square test.

## Results

A total of 305 of 317 respondents (96.2%) answered the closed question on provision of HPV vaccination at sexual health clinics, and 13.8% (42) strongly agreed, 33.8% (103) agreed, 26.6% (81) disagreed, 17.0% (52) strongly disagreed, and 8.9% (27) could not decide. Of these, 80% (244) of parents explained their viewpoint (open question). Parents who had indicated that they could not decide whether they agreed or disagreed with the concept were less likely to offer any explanation. The responses to the open and closed questions were cross-tabulated (Table 1). More positive views largely accorded with the agree/strongly disagree categories, as did the less positive with the disagree/strongly disagree categories. "Other" views were distributed across all categories.

**Table 1:** Cross tabulation of parental responses to open and closed questions addressing the topic of vaccination without parental consent

Parents views (**open question)**	A well-informed child could request vaccination at sexual health clinics without parental consent (**closed question**)					
	Don't know	Strongly disagree	Disagree	Agree	Strongly agree	Total (n)
Positive	3	0	0	68	28	99
Less Positive	4	41	46	6	0	97
Other	9	6	17	12	4	48
Total (n)	16	47	63	86	32	244

n is the number in each group

### Positive opinions (n = 99)

The largest group (n = 40) had a clear sense that children who were well informed should be able to give autonomous consent to vaccination. As one parent said, "*If my child had read all about it and is willing to take the responsibility for his choice, then I would be proud of him*" or, " *Our children are generally better informed and better able to make decisions than we are, they deserve to have this respected*". This did not necessarily imply approval of early sexual activity, but approval that the child was learning to make health decisions. Some linked this to recognition of a child's maturity. One said, "*A child mature enough to request vaccination does not need parental consent*." Three other parents made comments such as, *"Any child who is attending sexual health clinics would fall into a group who would benefit from this vaccine. They should be able to request it – if they are having sex and seem able to understand the issues*". A somewhat different perspective was presented by another group of parents (n = 23), who considered it important to protect children from parents who seemed not to act (in their view) in the child's best interests (the principle of beneficence). This was illustrated by the comment, "*Parents are not realistic about what their children do, and this may place their children at risk*", or, "*Too many parents have their moral views blinding them as to what their children actually get up to. Children should be protected, regardless of this"*, or *"It cannot be bad for a child to seek protection from a disease." *Another group (n = 27) indicated that the main factor was respect for a child's right to privacy. For example, *"A child might be in a sexual relationship and their parents don't know about it and they want to keep it quiet without their parents finding out." *Nine other parents framed their response simply in terms of children having a right to decide.

### Less positive opinions (n = 97)

Most of the opinions in this category reflected the view that parents felt, for one reason or another, that they should not be excluded from decision-making and that parental rights took precedence over the child's rights. Forty four made statements such as, *"Parents should always be informed" *or, *" My child doesn't request vaccination without parental consent because we are Asian" or*, "*I have been involved in decisions about all other vaccinations, so I should be informed about this one." *Some said that parents could not take responsibility for children if they did not know what the child was doing. A further group of 12 parents complained that parents' rights to make decisions on behalf of their children were being eroded as, "*Far too much responsibility for children's health and conduct is being taken out of parent's hands*, and, "*It's bringing a barrier between child and parents. Instead of parents discussing with their children, ethics, morals and values, the government is allowing the by-pass of parental authority and responsibility, while at other times, eg. for truancy, forcing unreasonable parental authority*." Related to this was a set of viewpoints (n = 18) emphasising the importance of parental guidance (principle of paternalism), "*because, after all, a child is a child, so it is best if he/she asks their parents for their permission as they have a lot of knowledge*."

One set of parents (n = 11) were explicit about parent's involvement in medical consultations related to HPV vaccination, making comments such as "*There could be a family history which may make it (the vaccine) unsuitable," *and "*In the event of side effects, how would parents know what to monitor?*" Three parents within this group added statements to the effect that *"parents should know about a child's sexual activity." *Another set of statements (n = 12) suggested that sexual health clinics were unsuitable and did harm (principle of maleficence) because moral issues were neglected and children given selected information, "*Children... will be informed but often times children are not the best judge to be able to weigh their decisions without parents (adults who have values)" *and "*Who assesses, (and how) whether the child is well informed *?" Nine of the 12 said that attending sexual health clinics encouraged sexual promiscuity. In the words of one, "*Unless parents and children are taught together about the risks of a sexually promiscuous life, I can see only a downward spiral of disease and social disintegration." *Three of the twelve claimed that it would be unnecessary to provide a vaccine at sexual health clinics if it was available in schools.

### Other views (n = 48)

Age, rather than maturity, was the deciding factor for 22 parents, who wanted clearer legal guidelines. This was indicated by comments such as, "*Children are minors. An age should be set for everything, drinking, sex etc and be the same*." This statement reflected a sense of confusion about different legal ages of consent for these activities. Some parents apparently disagreed with the current legal framework that allows under-16s to be treated, for example, *"Children over the age of 16 – yes, but younger children should not be taking medical advice without a parent." *The remainder (n = 26) wanted children and parents to work things out together, but their statements were normative rather than a rejection of adolescent autonomy. For example, one said, *"A child should discuss such issues with a parent" *and another, "*I wouldn't like my daughter to make a life-changing decision without being able to talk to me."*

Socio-demographic characteristics of the 244 parents were compared (Table 2). Parental age, receipt of free school meals and religion were not significantly associated with views on consent, but there were significant differences between ethnic groups, and White and Black Caribbean parents were more supportive of adolescent autonomy. Parents who had concerns about sexual health clinics were less likely than those with favourable views to agree to future HPV vaccination, even with parental consent (67% versus 89%; chi square: p < 0.001).

**Table 2:** Socio-demographic profiles of parents holding different views on HPV vaccination without parental consent.

	**Vaccination without parental consent**				
		
**Characteristic**	**Positive % (No)**	**Less Positive (%) No**	**Other % (No)**	**Total % (No)†**	p value*
Parental Age (years)					
≤30	47.1 (8)	41.2 (7)	11.8 (2)	7.1 (17)	
31 – 40	36.4 (40)	45.5 (50)	18.2 (20)	46.2 (110)	
> 40	42.3 (47)	35.1 (39)	22.5 (25)	46.6 (111)	0.5
Child receives free school meals					
No	45.5 (80)	36.9 (65)	17.6 (31)	73.6 (176)	
Yes	30.2 (19)	46.0 (65)	23.8 (15)	26.4 (63)	0.1
Religion					
None	46.3 (19)	36.6 (15)	17.1 (7)	17.1 (17)	
Catholic	32.7 (16)	32.7 (16)	36.2 (17)	20.4 (49)	
Protestant	44.2 (50)	41.6 (47)	14.2 (16)	47.1 (113)	
Muslim	33.3 (6)	50.0 (9)	16.7 (3)	7.5 (18)	0.2
Ethnic Group					
White	45.5 (75)	32.1 (53)	22.4 (37)	67.6 (165)	
Black Caribbean	39.1 (9)	47.8 (11)	13.0 (3)	9.4 (23)	
Black African	27.8 (5)	50.0 (9)	22.2 (4)	7.4 (18)	
India Sub-continent	28.6 (8)	57.1 (16)	14.3 (4)	11.5 (28)	
Others‡	20.0 (2)	80.0 (8)	0.0 (0)	4.1 (10)	0.03

† Total varies due to occasional missing data * Pearson's chi square

‡ eg. Chinese, Arab

## Discussion

In the UK there is a high teenage pregnancy rate and an epidemic of STIs [19]. The high and increasing prevalence of HPV [20] among young women is probably due to high-risk behaviour and biological immaturity of young adolescents exposed to infection [21]. The government's sexual health strategy promotes expansion of sexual health services and a determined effort to make these accessible to under-16s [22], often through avenues outside the clinical setting [23]. If projections about parental acceptability of vaccination are correct, it can be anticipated that, if parental consent is mandatory, at least 20% of under-16s will not be vaccinated against HPV. Strategic decisions will be needed on whether to try and optimise coverage by making the vaccine available without parental consent, and whether this would provoke negative reactions from parents.

In trying to answer these questions, it becomes obvious how little research (as opposed to media coverage) has been conducted, either in UK or elsewhere, on the acceptability to adults of policies allowing under-16s to be treated without parental consent [24-26]. Our research indicates strong, but divided, views on medical intervention without parental consent as well as confusion about the legal framework governing parental responsibility. Parents with favourable views referred spontaneously to children's ability to understand and process information, although it remains unclear precisely how they interpreted "well-informed". They argued from an ethical perspective in support of adolescent autonomy as a developmental, age-independent process of separation and detachment from the family [12] and this is consistent with allowing a competent adolescent to access confidential sexual health care. In reality, the right to treatment is still mediated by an adult, but it is a health professional instead of the parent. This role is approved by parents who argue that this protects vulnerable adolescents, but is challenged by other parents who question whether health professionals can judge what is in a child's best interests. Their views corresponded to a more recent definition of autonomy that emphasises maintaining "family connectedness" [12]. Largely based on US studies, family support is considered to encourage healthy development and to result in fewer risky behaviours [27-29] but is inconsistent with the principles of confidential adolescent sexual health provision. This is not to say that parents who want to be involved in health decisions would stand in the way of appropriate preventive or treatment strategies such as HPV vaccination, but that they want to be involved in the decision. We previously reported that 74% of parents intended to make a joint decision about HPV vaccination with their child and this was associated with support for the vaccine [9]. In the current analysis, we further demonstrate that some parents saw no need for the vaccine to be available at sexual health clinics if it could be provided in schools. For this reason, in the UK, a high HPV vaccine coverage may be achievable through a school-based strategy, as it was for adolescent hepatitis B vaccination [30], because parental consent will be sought.

The ethical question remains, however, of the right of adolescents to request the vaccine if, as minors, they subsequently become sexually active without their parent's knowledge and their parents earlier had declined consent. As the principle of adolescent autonomy is legally recognised in the UK, logically it should include the right to HPV vaccination [31]. In the Gillick case, the Law Lords focused on the issue of consent rather than a notion of parental rights or powers. In fact the courts held the view that parental rights did not exist, other than to safeguard the best interests of the minor [32]. One scenario would be to argue that HPV vaccination is different from other sexual health interventions because effective cervical screening programmes provide a safety net for adolescents who subsequently develop cervical intraepithelial lesions. This argument could not be applied with respect to genital warts if a quadrivalent vaccine was in use. This is an important consideration because in 2001 there were 32,185 diagnoses of genital warts in females, 29% of which were in those under 20 years of age [33]. Another approach would be to vaccinate under-16s without parental consent within a catch-up programme that was accessible to this age group. Catch-up programmes are expensive and it is not clear whether vaccination will benefit women already exposed to HPV [3] but they are quite likely to be adopted as an interim measure. It would be important to ensure that adolescents were aware of catch-up programmes as those who have not been vaccinated may feel anxious if most of their peers received the vaccine.

There are a number of limitations to this study, notably with the use of semi-qualitative data taken from surveys [18], which is intended to provide insights not conveyed by the quantitative data. Being partly quantitative, biases in the survey could be reflected in the analysis, and with a relatively low response rate, the results cannot be assumed to represent all parents' views. A greater limitation is that semi-qualitative data are not detailed and do not provide the more informative type of data that can be gained from in-depth qualitative approaches. Nevertheless, our study provides some insights into the need for policy makers to engage more effectively with parents on sexual health matters. Parents rejecting the principle of adolescent autonomy were more opposed to adolescent HPV vaccination *per se*. For adolescents, access to health care is always mediated by gate keepers such as parents, health providers and communities and it is vital not to undermine efforts to make it easier for them to obtain the health care they require. A public debate is needed to allow all perspectives to be considered and to help parents and policy makers to understand the ethical issues that underpin adolescent autonomy, confidentiality and privacy [34], as well as the skills required to talk to children and promote adolescent self-regulation [35]. The doctor-patient relationship is the primary focus of ethics in medicine, and clinicians must be prepared to discuss these issues with adolescents and their parents [36].

## Conclusion

Although recognising its benefits, some parents might disapprove of HPV vaccination without parental consent. This could pose a dilemma in countries where adolescent sexual autonomy is legally recognised and might contribute to the view that the vaccine encourages sexual promiscuity. Such concerns would need to be openly addressed to avoid misunderstanding that the purpose of vaccination is prevention of cervical cancer.

## Competing interests

LB and HCK have undertaken consultancies and received travel grants from GlaxoSmithKline (GSK) which manufactures Cervarix. In addition HCK has received research funds for clinical trials of this vaccine. GSK has had no part in this research study.

## Authors' contributions

LB contributed to all stages of the research and analysis; SAR contributed to the design and analysis; HCK reviewed the manuscript. All authors read and approved the final manuscript.

## Pre-publication history

The pre-publication history for this paper can be accessed here:


